# Extracts from the Records of the Atlanta Medical Society

**Published:** 1858-04

**Authors:** V. H. Taliaferro

**Affiliations:** Secretary


### ARTICLE. III.

*Extracts from the Records of the Atlanta Medical Society.* By V. H. TALIAFERRO, M. D., Secretary.

Dr. J. G. WESTMORELAND, reported a case of Puerperal Convulsions, in a primapara, at the expiration of the seventh month, of Utero-gestation. Dr. Westmoreland reports, that when he first saw this case, there had been slight uterine contractions, according to the imperfect history received, for five hours. Vertigo, with some uneasiness of the chest, occurred ten hours before the symptoms of uterine contraction, succeeded by head-ache, which last symptom continued up to the time of his first visit, at 12 o'clock at night of 14th March; one-fourth of a grain of acetate Morphine was given, with the view of preventing the premature delivery, but from the irritability of stomach, was rejected. It was repeated, however, until the pains were arrested, in a few hours, and the patient felt comfortable at 4 A. M. At 7, three hours after leaving, was summoned in haste, and found her just recovering from the second convulsion. Chloroform was obtained as speedily as possible, and administered every hour, in the dose of 15 drops; and inhaled when symptoms of a convulsion were discovered, generally with very prompt relief. The uterine contractions were moderate and ineffectual; the os uteri only slightly dilated and rigid. Patient stupid through the day—not rational—delivered at 10 P. M., nearly twenty-four hours from my first visit. Another convulsion came on shortly after delivery. Symptoms of their return with great prostration, were effectually met by stimulants and the inhalation of Chloroform. In six or eight hours after the third convulsion, consciousness was restored, and the case has progressed favorably up to this time, (18th.)

My conclusions are, that in this case, the morphine given to arrest the uterine contractions, added to the liability of convulsions, and might probably have been happily substituted by the Lancet, inasmuch as the opiate failed to control the labor, and as all the circumstances of the case seem now to justify the opinion, that the fœtus was dead in utero, before the contraction of the uterus commenced.

Dr. J. W. Jones, Sr., said:—While he was conscientiously constrained to dissent in part, from the practice in this case, yet he was highly gratified by the frankness and candor of the gentleman with whom he was compelled to differ. That he regarded the report of Dr. W., as highly interesting, and as well calculated to elicit the contrast between present and past Pathology and Therapeutics. He believed that it was of late, too generally the practice of the profession, to substitute anodynes and anesthetics, for less pleasant, but more reliable remedies. That the former, however popular, were in too many instances not appropriate to the indications. He was opposed to the placebo practice; was an advocate of the Lancet in genuine Eclampsia, and seldom administered opiates or anodynes, prior to, or in lieu of venesection in such cases, and believed himself well sustained by both ancient and modern authorities. That opiates when thus employed, should be administered per *anum*, rather than per orem. His experience justified the belief that puerperal convulsions in this country, were more frequent in the robust and plethoric, than in those of opposite constitution and habit. He regarded the disease (puerperal convulsions) as originating in most cases, either in nervous excitability, or hyperemia, or both. That the neuro-hæmatic, etiology, was the middle, the rational and safe ground. He did not consider hysteria, catalepsy, tetanus, &c., as by any means analogous to the disease under consideration. That withholding the lancet in the treatment of puerperal convulsions, was an exception to a general or well-established practice, and rarely justifiable. He did not bleed in every case of eclampsia. Thought his position sustained by both ancient and modern authority. He was not opposed to the timely use of hypnotics and antispasmodics as auxiliaries, but was disposed to rely mainly upon blood-letting and cathartics, as the best prophylactic and curative means in such cases. That the brandy in this case was as much a derivative as was the cathartic, or the sinapisms, and was therefore equally appropriate. The The modus operandi of each, being on the principle *ubi irritatio ibi affluxus.*

DR. LOGAN, considered puerperal convulsions one of the most interesting pathological manifestations which could be presented to the physician, and especially so to him at present, as he believed that a radical error prevailed in reference to the true character of the *condition* from which the disease resulted, and, as a necessary consequence, in the *treatment* of the affection.

He stated, as the result of his observation, that puerperal convulsions were generally found in females of highly organized nervous systems, and who were peculiarly susceptible to irritability of that system, and not among the plethoric and robust—in those who had no superabundance of blood, and did not, therefore, require the active depletion which had been so generally resorted to in the treatment of this affection—rather than in those of an opposite constitution. He did not attach so much importance as medical men generally, to the idea of cerebral congestion, as the cause of this disease, but believed its true pathology to be a peculiar nervous erethism, *dependent upon* the irritable condition of that portion of the nervous structure in connection with the uterus, in consequence of the peculiar condition of that organ during the puerperal state, and thus *predisposing* the female to a convulsive attack upon the addition of an *exciting cause*, which is most usually the occurrence of uterine contraction.

In the treatment of the convulsions, it was considered a matter of the greatest importance to deliver the female, and as aiding this process, in some instances, blood-letting might be beneficial, but as a general rule, he not only considered it unnecessary, but injurious. He had seen the case under consideration, and could state that it had been attended with the most alarming prostration, rather than excitement, and could not possibly have been benefitted by blood-letting, and had required stimulation instead of depletion, and afterwards under the influence of chloroform, the convulsions had been controlled until delivery was effected.

To show still more decidedly the fact, that the convulsions were not dependent upon that condition requiring blood-letting for its removal, after the contents of the uterus were expelled, and during the most extreme depression (the use of the chloroform having been suspended) the convulsions returned with great violence, but were again controlled by the chloroform in connection with brandy, until reaction was fully established, when there was a final disappearance of the eclampsia.

Dr. W. F. Westmoreland fully concurred with Dr. Logan, as regards the character and treatment of the affection. Regarded the congestion of the brain which exists in some cases as the result, and not the cause of the convulsions. Thinks that much mischief has resulted from the too popular idea, that, in puerperal convulsions, there is always a congestion of the brain prior to the access, and that to relieve the patient, copious blood-letting is essential. He mentioned a case which came under his observation, where the lancet had been resorted to, until an incredible amount of blood had been taken from the arm without arresting the convulsions, and which was afterwards promptly and entirely relieved by the administration of chloroform. Thought, that if there was no other evidences, the treatment which has been found most successful, and which no one would think of employing in congestion of the brain, would be sufficient to controvert the popular opinion of plethora and congestion.

Dr. Darnall—said, my usual course, when called on to see a patient in the latter period of pregnancy, complaining of the symptoms reported by Prof. J. G. Westmorland, viz: Giddiness, and pain in the head, is about as follows: If there is fever, or if the superficial veins fill up readily, and feel tense, (which can be easily determined by grasping the wrist of the patient firmly with your hand,) I do not hesitate to use the Lancet, with more or less freedom, according to the urgency of the symptoms. If there is much plethora, a *free* bleeding will, generally, be necessary to relieve the patient. No dangerous debility need be apprehended from the loss of blood, if the patient is able to sit up during its flow; for as long since remarked by Dr. Marshall Hall, "as soon as the quantity lost, reaches the point of toleration, symptoms will present themselves, which will induce the operator to arrest its flow"—I mean incipient syncope. But Sir, I do not wish to be understood, as advising this course in *all* cases. We sometimes meet with ladies in this interesting situation, whose condition is precisely the reverse of plethora—they have all the symptoms of anemia. Instead of direct depletion, they require anodynes, stimulants, &c., &c.

In regard to the treatment of Puerperal Eclampsia, the Lancet stands in my estimation, pre-eminently at the head of the list of therapeutic agents; because, with it you can rapidly diminish the volume of the blood, and save your patient from the horrors of effusion, or extravasation. There may be cases where venesection may be contra indicated; yet, candor compels me to say that I have not seen such an one. The cases I have seen, with but few exceptions, have been young women in their first pregnancy, with a considerable degree of plethora. I can now only call to mind the case alluded to by my friend, Prof. W. F. WESTMORELAND, and one other case. These were both small delicate ladies, in whom the nervous temperament predominated. Both of these cases lost blood enough–one was relieved entirely by it alone—the other, (the case referred to by Prof. W. F. W.) only partially. Chloroform was then given in doses of twenty or thirty drops, every half hour, for an hour or two, after which, she had no return of convulsions, and was readily and safely delivered of a dead fœtus, in less than twenty-four hours, after their cessation, without the aid of Instruments.

The beneficial effect of Chloroform in this case, was so palpable, that under *similar* circumstances, I would not hesitate to use it again.

Dr. TALIAFERRO, reported the following:

*Gentlemen of the Atlanta Medical Society*:—I propose presenting to you this evening, in a hasty manner, some of the post mortem appearances of a case recently examined by Prof. W. F. Westmoreland and myself, with some few other medical gentlemen. The subject was that of a female who had died after protracted illness, but in consequence of having been attended by another physician whom we did not consult, I am unable to give you a correct history of her symptoms during the period of her confinement.

The following, however, we gathered from the nurse (her mother.)—Age about 20. For some considerable length of time, had been in delicate health, suspected to have had syphilis, and had aborted.

The principal seat of pain we were told, had been in the lower part of the abdomen. The discharges from the bowels and the bladder, had been attended with much pain and difficulty. The urine frequently presenting an appearance, as if intermixed with fœcal matter, and very offensive.

Aside from the above, she had been addicted to the deplorable practice of habitual and excessive snuff using.

The appearance of the interior portion of the stomach of this unfortunate female, I opine, could it have been exhibited to many of our *refined* and *aristocratic* snuff dippers, would have effectually eradicated their cultivated morbid propensity. Upon laying open the abdomen, we at once perceived extensive and serious disease of the bladder and surrounding tissues.

Dr. Westmoreland, with a great deal of care, succeeded in taking out entire, the bladder, the womb and ovaria, vagina and rectum; the specimen of which I have the pleasure of presenting to you. Care was necessary in the extraction of these organs from the abdominal cavity, from the fact of extensive adhesion, and destruction of tissue by ulceration.

The greater and superior portion of the bladder, as you will perceive, has been destroyed by ulceration, which was doubtless syphilitic. The rectum also, was the seat of extensive ulceration, which resulted as you see, in a larger opening through its coats. The large openings in the rectum and bladder communicate with an opening, just beneath one of the broad ligaments and fallopian tube. Above the perforation of the rectum, there is an extensive stricture of the intestine, (barely admitting an ordinary size bougie) and an enormous thickening of its walls. The uterus we found not diseased.

Our examination next extended to the lungs, where we found no abnormal condition, save adhesion and chronic inflammatory action of the lower portion of the right lobe.

The stomach was next examined, and no disease of an organic character detected, but found it partially filled with a thick, dark fluid, which we had every reason to believe, received its color and consistence from its intermixture with snuff. A small quantity of the fluid taken in a cup, very soon deposited a thick sediment, the character of which was unmistakeable.

The entire interior surface of the stomach was thickly lined with a heavy sediment of snuff.

We were told that the subject lay with it in her mouth continually, during the period of her confinement.

This case presents some points of no little interest. In the first place, that life could have been prolonged until the bladder and rectum were so extensively diseased.

In the second place, that a delicate female, much debilitated and emaciated by a loathsome and chronic disease, could have so accustomed her system to the influence of tobacco, as not to have died alone from the amount of snuff her stomach contained at the time of examination.

